# Structure–Electrical Property Relationships of Spike-Structured Conductive Silicone Interfaces for Wearable Trigeminal Microcurrent Stimulation in Electroceutical Devices

**DOI:** 10.3390/polym18121473

**Published:** 2026-06-12

**Authors:** Tae-Hun Kim, Ji-Hong Bae, Jiwon Cheon, Eun-Ji Kim, Eunsoo Kim, Young-Suk Jung

**Affiliations:** 1 Corporate Research Institute, PTBRO Inc., Busan 46317, Republic of Korea; kth763321@naver.com (T.-H.K.); jiroro33@naver.com (J.C.); hoi421@naver.com (E.-J.K.); 2 College of Pharmacy, Research Institute for Drug Development, Pusan National University, Busan 46241, Republic of Korea; jhbae@pusan.ac.kr; 3 Research Institute for Natural Product-Based Food and Medicine, Pusan National University, Busan 46241, Republic of Korea

**Keywords:** conductive silicone interface, spike-structured electrode, trigeminal microcurrent interface, volume-resistive conductive elastomer, geometry-derived electrical descriptor, transient electrical response

## Abstract

Conductive silicone interfaces are promising polymeric materials for wearable bioelectronic systems because they combine electrical continuity with elastomeric compliance, environmental durability, and moldability. In low-voltage wearable microcurrent interfaces, however, functional performance is governed not only by intrinsic material conductivity, but also by conductive network continuity, molded geometry, interfacial contact, and transient electrical response. In this study, we developed a spike-structured conductive silicone interface using a commercially available electrically conductive two-component silicone rubber and investigated its structure–electrical property relationships as a volume-resistive polymer interface. The interface consisted of a conductive silicone body with protrusions 7 mm in height and 3.6 mm in diameter, supported by a 1 mm base layer and electrically integrated through an Ag-paste-connected upper conduction region. Using a representative electrode-level resistance of 47.08 Ω, the geometry-derived apparent interfacial resistive response was estimated as 18.0 Ω·cm for the three-spike configuration and 24.0 Ω·cm for the four-spike configuration. The corresponding effective conductive areas were 0.305 cm^2^ and 0.407 cm^2^, respectively, giving analytical current-density amplification factors of 9.82 and 7.37 relative to a planar 3 cm^2^ reference interface. Positional resistance mapping yielded an overall mean resistance of 47.80 ± 4.57 Ω, indicating acceptable electrical reproducibility across the structured conductive silicone interface. In addition, oscilloscope-based transient response analysis under a 5 V, 1 kHz square-wave input showed that the conductive silicone interface maintained the overall pulse waveform while showing a modest reduction in overshoot from 3.4 ± 0.1% to 2.7 ± 0.1%, with FFT traces used as qualitative waveform-monitoring displays. Formulation-dependent comparison further showed that increasing the silicone-rich fraction increased the measured resistance from 105 Ω to 145 Ω, whereas increasing conductive carbon loading reduced resistance but aggravated surface transfer. These results show that the conductive silicone interface functions not simply as a soft conductor, but as a volume-resistive, geometry-defined current-transfer medium whose behavior is governed by the coupled effects of conductive network formation, spike architecture, electrode-level resistance, and transient pulse response. This study provides a practical materials/interface design framework for spike-structured conductive silicone electrodes in wearable bioelectronic and electroceutical devices.

## 1. Introduction

Wearable bioelectronic systems have advanced rapidly as soft, skin-interfaced platforms for physiological monitoring, non-invasive stimulation, and emerging electroceutical applications. In these systems, the electrode is not merely a passive conductor, but a functional interface that must simultaneously provide electrical continuity, mechanical compliance, conformal skin contact, and operational stability during repeated use. Soft dry electrodes are particularly attractive because they can overcome several limitations of conventional gel-based electrodes, including dehydration, progressive signal degradation, and user discomfort during prolonged application. Consequently, the performance of wearable dry electrodes is governed not only by material conductivity, but also by interfacial mechanics, structural reproducibility, electrode geometry, and device-level integration [1,2,3,4,5,6,7,8].

Among the material platforms investigated for soft electrodes, conductive elastomers and silicone-based conductive systems have received considerable attention because they combine deformability with electrical transport through percolated conductive networks. Silicone-based materials are especially relevant because they provide elasticity, chemical stability, environmental durability, and established processing routes for molded and microstructured components [9,10,11,12,13,14,15,16]. In parallel, recent soft-electrode studies and reviews have reported advanced platforms such as liquid-metal-based stretchable conductors, conductive hydrogel electrodes, PEDOT:PSS- or conducting-polymer-based interfaces, carbon-nanomaterial-based skin electrodes, and porous graphene-based dry electrodes. These systems offer important advantages such as high stretchability, conformal contact, reduced motion artifacts, low interfacial impedance, and improved electrochemical coupling [17,18,19,20,21]. However, many of these systems are primarily optimized for biosignal recording, ultrastretchable electronics, hydrated low-impedance contact, or coating-based electrochemical interfaces. For reusable wearable microcurrent interfaces based on molded conductive silicone, the engineering challenge is therefore not simply to maximize conductivity or softness, but to balance conductive network continuity, volume-resistive behavior, dry elastomeric robustness, reproducible geometry, contact stability, and compatibility with low-voltage operation.

Structural design is also a critical factor in determining the performance of dry and soft electrodes. Microstructured, compliant, or patterned electrode surfaces can improve effective contact, reduce motion-related instability, and modulate current transfer at the interface [22,23,24,25,26]. In wearable trigeminal microcurrent systems, this structural requirement becomes particularly important because the electrode must maintain stable contact on curved and mechanically sensitive facial or craniofacial regions while providing localized current transfer through a soft and reproducible contact geometry [27,28,29,30,31,32,33]. In this context, spike-structured conductive silicone interfaces are practically attractive because they can combine dry elastomeric contact, molded geometric reproducibility, and localized current-transfer characteristics in a single soft polymeric component. However, the structure–electrical property relationship of molded conductive silicone interfaces for such low-voltage wearable microcurrent systems remains insufficiently clarified from a conductive silicone materials and device-integration perspective.

Another important point is that the conductive silicone electrode in a wearable microcurrent device operates as the terminal interfacial material of a low-voltage system rather than as an isolated bulk conductor. Under such conditions, practical performance depends not only on intrinsic resistivity, but also on how the volume-resistive conductive silicone body, spike geometry, and contact configuration affect circuit-defined pulse transfer while preserving elastomeric compliance and structural reproducibility. This perspective suggests that conductive silicone in wearable microcurrent systems should be interpreted not simply as a soft conductor, but as a volume-resistive, geometry-defined current-transfer medium integrated into a low-voltage device architecture [34,35,36,37].

In this study, we developed a spike-structured conductive silicone interface for wearable trigeminal microcurrent devices and investigated how electrode geometry, geometry-derived electrical descriptors, conductive network behavior, electrode-level resistance, and transient pulse response are interrelated. Commercially available electrically conductive two-component silicone rubber was employed as the primary conductive material, and the resulting interface was analyzed through structure-based electrical interpretation, positional resistance mapping, transient electrical response analysis, and formulation-dependent comparison. The central hypothesis was that the functional behavior of the conductive silicone interface is governed not by conductivity alone, but by the coupled effects of conductive network formation, volume-resistive behavior, spike architecture, compliant contact, and transient electrical stability. The novelty of this work lies not in the discovery of a new conductive filler chemistry or in the physiological validation of trigeminal stimulation, but in the structure–electrical interpretation of a molded spike-structured conductive silicone interface as a reproducible, volume-resistive, geometry-defined current-transfer medium for low-voltage wearable microcurrent devices. On this basis, the present work aims to provide a practical materials/interface design framework for conductive silicone-based wearable bioelectronic and electroceutical interfaces.

## 2. Materials and Methods

### 2.1. Materials

A commercially available electrically conductive silicone rubber, ELASTOSIL^®^ R 573/70 A/B (Wacker Chemie AG, Munich, Germany), was used as the primary conductive material for fabricating the spike-structured conductive silicone interface. According to the manufacturer, this material is a two-component, addition-curing, electrically conductive high-consistency silicone rubber intended for conductive molded products and components. The product is supplied as an A/B system mixed at a ratio of 1:1 and is described as a low-volume-resistivity conductive silicone material within a high-consistency silicone rubber platform [38,39,40]. Because the detailed filler composition and compounding formulation are proprietary, the material was treated in this study as a commercial conductive silicone rubber containing a distributed carbon-based conductive network, rather than as a newly synthesized conductive composite.

For material-positioning purposes, a conductive liquid silicone rubber (LSR), SILASTIC™ HV 1523-30 Liquid Silicone Rubber Part A&B Kit (Dow, Midland, MI, USA), was considered as a representative commercial conductive LSR comparator. According to publicly available manufacturer information, this material is a two-part, 1:1-mix conductive LSR designed for electrically conductive moldings and electrical stress-control applications. Supplier documentation reports a hardness of approximately 30–35 Shore A, high elongation at break, and volume resistivity of approximately 80 Ω·cm in electrical-grade LSR selection data [41,42]. In the present study, this material was used only as a catalog-based commercial comparator representing a softer conductive LSR class. It was not fabricated into the same spike-structured electrode and was not tested as a direct experimental control under identical conditions. Therefore, no direct performance superiority is claimed from this catalog-based comparison.

**Scheme 1:**
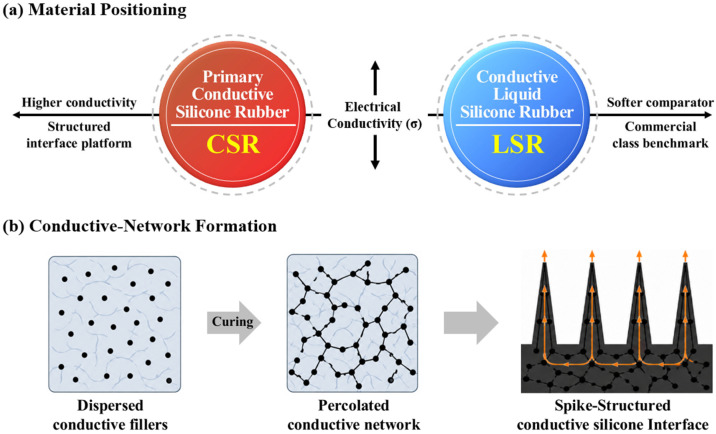
Conceptual material positioning and conductive-network formation of the conductive silicone system used for the spike-structured wearable interface. (**a**) Material-class positioning of the conductive silicone rubber (CSR) and conductive liquid silicone rubber (LSR). (**b**) Schematic illustration of conductive-network formation in the conductive silicone matrix. Dots indicate conductive fillers, connected lines indicate the percolated conductive network, and arrows indicate the conceptual material-positioning direction or current-transfer pathway.

A silver paste layer was used to establish electrical connection between the conductive silicone body and the external circuit. The Ag paste was introduced only at the PCB-side contact region to provide a stable electrical pathway between the cured conductive silicone body and the circuit board. This was necessary because direct mechanical contact between cured conductive silicone rubber and PCB pads may lead to unstable contact resistance during repeated handling, deformation, or assembly. The contact-facing spike region itself was formed entirely from conductive silicone, and no rigid metallic spike or embedded metal needle was inserted into the contact region. The conceptual material positioning used in this study is summarized in Scheme 1.

### 2.2. Fabrication Strategy of the Conductive Silicone Electrode

The conductive silicone electrode was fabricated through material preparation, homogenization, degassing, molding, thermal curing, and electrical integration. Because the exact formulation and compounding details of the commercial conductive silicone are proprietary, the present work focused on structure–electrical property interpretation rather than disclosure of formulation chemistry. Accordingly, the conductive elastomer was treated as a silicone matrix containing a distributed carbon-based conductive network, consistent with general design principles reported for conductive filler-network-based elastomers and soft conductive materials [43,44,45,46]. After curing, electrical continuity was interpreted as arising from the pre-existing conductive filler network within the commercial conductive silicone rubber, which is maintained through homogenization, degassing, molding, and curing under the recommended processing conditions.

**Scheme 2:**
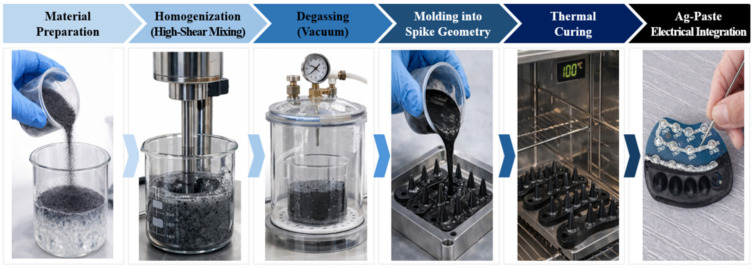
Schematic fabrication route of the spike-structured conductive silicone interface, including material preparation, high-shear homogenization, vacuum degassing, molding into spike geometry, thermal curing, and Ag-paste electrical integration.

The fabrication sequence consisted of (i) material preparation, (ii) homogenization by high-shear mixing, (iii) vacuum degassing, (iv) molding into the spike-shaped geometry, (v) thermal curing, and (vi) Ag-paste electrical integration. As summarized in Scheme 2, these steps were used to obtain a reproducible spike-structured conductive silicone interface with electrical connection to the circuit board. Because the primary material was a commercial two-component conductive silicone rubber, the A/B mixing ratio was fixed at the supplier-recommended 1:1 ratio. Therefore, the sample preparation optimization focused on processing variables that directly affect electrode reproducibility, including mixing uniformity, bubble removal, mold filling, curing completeness, and electrical integration with the Ag-paste contact layer.

The electrode architecture followed a Silver Spike Point-inspired concept and consisted of a conductive silicone base layer supporting multiple protrusions. Each protrusion had a height of approximately 7 mm and a representative conductive diameter of approximately 3.6 mm, and the supporting base layer had a thickness of approximately 1 mm. Two structural configurations were designed: a three-spike structure and a four-spike structure. In both cases, the protrusions were electrically connected in parallel through the same continuous conductive silicone body, giving an effective current-path length of approximately 8 mm. The spike height, spike diameter, and base-layer thickness were selected to maintain molded structural fidelity, stable contact geometry, and compatibility with the wearable device housing. The three-spike and four-spike layouts were selected as practical structural variables for comparing effective conductive area and geometry-dependent current-transfer behavior under otherwise fixed material and dimensional conditions.

**Figure 1:**
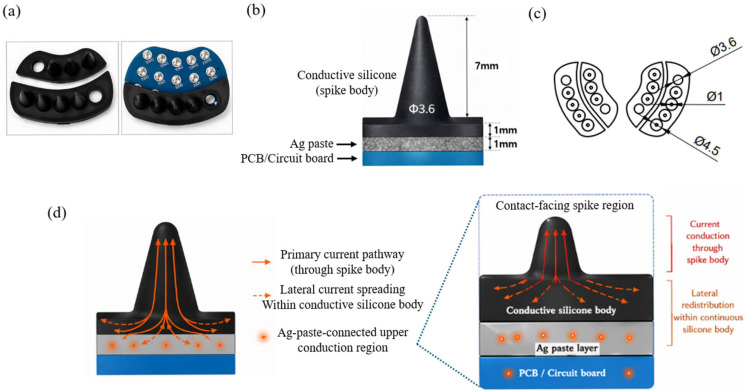
Structural design of the spike-structured conductive silicone interface. (**a**) Overall interface geometry. (**b**) Dimensioned side-view schematic showing the conductive silicone spike body, Ag-paste layer, and PCB/circuit board. (**c**) Top-view comparison of the three-spike and four-spike layouts. (**d**) Cross-sectional schematic of current transfer through the conductive silicone body and the Ag-paste-connected upper conduction region.

The detailed structural design of the fabricated interface is shown in Figure 1. Figure 1a presents the overall interface geometry, Figure 1b shows the dimensioned side-view schematic, and Figure 1c summarizes the top-view layouts of the three-spike and four-spike configurations. Figure 1d illustrates the cross-sectional current-transfer pathway through the conductive silicone body and the Ag-paste-connected upper conduction region. In this configuration, circuit-defined current is introduced from the PCB-side contact region through the Ag paste and then transferred within the continuous conductive silicone body toward the spike region. This illustration is intended as a conceptual representation of current transfer through the structured conductive silicone interface, not as a direct field-mapping result or PCB-resolved simulation.

### 2.3. Analytical Framework for Electrical Parameter Estimation

To analyze the structure-dependent electrical behavior of the conductive silicone interface, the electrode was described using a geometry-based resistance model. The protrusion diameter used for the effective-area calculation was fixed at 3.6 mm, corresponding to a radius of 0.18 cm, and the effective current-path length, L, was defined as 0.8 cm from the sum of the 7 mm protrusion height and the 1 mm base thickness. For comparison of geometry-dependent current-transfer behavior, a planar reference area of 3 cm^2^ was used.

The cross-sectional area of a single protrusion was defined as

(1) $$A_{\mathrm{single}}=\pi r^{2}=\pi {\left(\frac{d}{2}\right)}^{2}$$

where d is the protrusion diameter and r is the protrusion radius.

Because the protrusions act as parallel conductive pathways within the same continuous conductive silicone body, the total effective conductive area was defined as

(2) $$A_{\mathrm{eff}}=nA_{\mathrm{single}}$$

where n is the number of protrusions.

The apparent interfacial resistive response of the structured electrode assembly was estimated using

(3) $$\rho_{\mathrm{app}}=\frac{R_{\mathrm{rep}}A_{\mathrm{eff}}}{L}$$

where $R_{\mathrm{rep}}$ is the representative electrode-level resistance used for structure-based analysis. In this study, $R_{\mathrm{rep}}$ was a measured representative resistance value obtained under the defined electrode-level measurement configuration. In contrast, $\rho_{\mathrm{app}}$, $A_{\mathrm{eff}}$, and $\Gamma_{J}$ were geometry-derived analytical descriptors calculated from the measured resistance and predefined structural dimensions. Therefore, these parameters should not be interpreted as independently measured bulk resistivity, directly measured current density, experimentally mapped current distribution, or PCB-resolved field-simulation results.

For conceptual comparison, the idealized resistance of a homogeneous conductive path may be written as

(4) $$R_{\mathrm{ideal}}=\frac{\rho_{\mathrm{bulk}}L}{A_{\mathrm{eff}}}$$

This relationship was introduced only to distinguish idealized bulk conductive behavior from the apparent electrode-level response of the structured conductive silicone interface.

To provide an effective-area-based interpretation of geometry-dependent current-transfer behavior, the local current density was described as

(5) $$J=\frac{I}{A_{\mathrm{eff}}}$$

and the current-density amplification factor relative to a planar reference interface was written as

(6) $$\Gamma_{J}=\frac{J_{\mathrm{spike}}}{J_{\mathrm{planar}}}=\frac{A_{\mathrm{planar}}}{A_{\mathrm{eff}}}$$

where $A_{\mathrm{planar}}$ is the planar reference area. This comparison was intended as an analytical descriptor under the same total-current assumption, rather than as a direct measurement of current density or spatial field distribution.

In addition, the overall device current path was conceptually written as

(7) $$I(t)=\frac{V_{\mathrm{drive}}(t)}{R_{\mathrm{series}}+R_{\mathrm{silicone}}+Z_{\mathrm{interface}}(\omega )}$$

where $V_{\mathrm{drive}}(t)$ is the circuit-defined driving voltage, $R_{\mathrm{series}}$ represents fixed series elements of the control circuit, $R_{\mathrm{silicone}}$ is the electrode-level resistance contribution of the conductive silicone interface, and $Z_{\mathrm{interface}}(\omega )$ represents a frequency-dependent interfacial contribution under practical contact conditions. This framework was introduced only to place the conductive silicone interface within the device-level current path of a low-voltage wearable microcurrent system. It was not used to claim human skin–electrode impedance measurement, electrochemical impedance spectroscopy, or physiological stimulation efficacy.

### 2.4. Electrical Resistance Evaluation and Supplementary Formulation Comparison

The electrical response of the conductive silicone interface was evaluated by positional resistance mapping and structure-based interpretation of apparent electrode-level behavior. Four contact positions were measured, and each position was tested in triplicate at room temperature using a digital multimeter under a fixed probe-contact configuration. Resistance was measured using a two-point probe configuration. Therefore, the measured values include contributions from the conductive silicone body, the Ag-paste-connected region, and the probe-contact interface.

For this reason, the measured values were interpreted as apparent electrode-level resistance values rather than intrinsic bulk resistivity values of the raw conductive silicone material. During measurement, the probe positions and contact configuration were kept constant to minimize contact-condition-dependent variation. The mean resistance at each position was used for positional mapping, while the representative electrode-level resistance used for geometry-based modeling was defined separately as a device-level analytical value. This type of evaluation is consistent with general considerations for dry electrodes and soft conductive interfaces, where reproducibility, positional variation, contact condition, and effective interfacial behavior are often more informative than nominal bulk conductivity alone [47,48,49,50].

The resistance data were reported as mean ± standard deviation, and the coefficient of variation was calculated to evaluate positional reproducibility across the fabricated conductive silicone interface. A supplementary formulation-dependent comparison was also performed to interpret conductive network behavior in the elastomer. In this comparison, the effects of the silicone-rich formulation condition and conductive carbon loading on the measured resistance and practical surface behavior were examined under the same measurement configuration. These observations were used to discuss the trade-off between electrical transport and practical interfacial usability in conductive silicone systems. The corresponding results are presented in Section 3.

### 2.5. Transient Electrical Response Measurement

To further evaluate the effect of the conductive silicone interface on transient pulse transfer, square-wave response measurements were performed using a RIGOL DHO924S digital oscilloscope (RIGOL Technologies Co., Ltd., Suzhou, China). A 5 V, 1 kHz square-wave signal was applied under a controlled measurement configuration, and the output waveform was recorded with and without the conductive silicone interface. The time scale was set to 20 μs/div.

The waveform shape, peak-to-peak voltage, edge transition, overshoot, and fast Fourier transform (FFT) display were monitored to examine the transient pulse-transfer behavior of the conductive silicone interface. Overshoot was evaluated from the transient peak relative to the steady plateau level of the square-wave response. The FFT display was used as a qualitative frequency-domain representation of the measured waveform and was not used for quantitative impedance extraction or harmonic attenuation analysis. This measurement was intended to provide conductive-silicone-centered transient response information. It should not be interpreted as full electrochemical impedance spectroscopy, human skin–electrode impedance characterization, current-distribution simulation, or physiological stimulation validation. Rather, the transient response analysis was used as an additional experimental method to examine how the conductive silicone polymer interface affects pulse transmission and rapid transient components under controlled measurement conditions.

### 2.6. Low-Voltage Device-Level Coupling

The developed conductive silicone interface was implemented in a low-voltage wearable microcurrent system based on a compact battery-powered circuit board comprising a microcontroller, MOSFET switching elements, and output channels connected to the conductive silicone contact interface. The power source was a 3.7 V-class Li-ion polymer battery with a rated capacity of 380 mAh, a charging voltage of 4.2 V, and an internal impedance of 200–260 mΩ.

Under this configuration, the conductive silicone was treated as the terminal interfacial material that transfers circuit-defined pulse signals toward the contact-facing spike region rather than as the current-generating element itself. Accordingly, the circuit-coupling description in this study was used only to define the role of the conductive silicone interface within the low-voltage device current path. It was not used to determine physiological stimulation efficacy, user response, or actual current delivered to human tissue. The device-level coupling interpretation based on this framework is discussed in Section 3.

## 3. Results and Discussion

### 3.1. Device-Derived Design Window of the Conductive Silicone Interface

The first important outcome of this study is that the conductive silicone in the present wearable microcurrent platform should be interpreted through device-derived interfacial constraints rather than through conductivity alone. The interface was required to satisfy four conditions simultaneously: low-voltage pulse transfer, localized interfacial current transfer, soft conformal contact, and stable reproduction of the spike geometry. These requirements distinguish the present system from conventional conductive rubbers, metallic conductors, or highly filled conductive pastes and define a practical interfacial window for wearable bioelectronic and electroceutical operation.

This design logic is reflected in the target interfacial window summarized in Table 1. The significance of this window is not that it reproduces the catalog bulk properties of any single raw material, but that it describes a device-relevant regime of interfacial function in which the electrode remains conductive enough for pulse transfer while preserving structural fidelity, contact stability, and practical wearability. Within this interpretation, the primary conductive silicone rubber and the softer conductive LSR comparator represent two distinct commercial material classes rather than two materials compared experimentally under identical spike-electrode fabrication conditions. In particular, ELASTOSIL^®^ R 573/70 A/B represents a higher-conductivity conductive silicone rubber platform positioned for structured current transfer, whereas SILASTIC™ HV 1523-30 represents a softer conductive LSR class with substantially higher reported volume resistivity. Thus, Table 1 should be interpreted as a materials-positioning and design-window framework rather than as a direct paired performance comparison.

Compared with recently reported soft-electrode platforms, the present conductive silicone interface is not positioned primarily as an ultrastretchable conductor, a hydrated low-impedance electrode, or a coating-based electrochemical interface. Rather, its significance lies in the use of a molded conductive silicone body as a reproducible spike-structured current-transfer medium in a wearable device. In this framework, dry elastomeric robustness, mold-defined geometry, positional resistance reproducibility, and transient pulse-transfer stability are emphasized as key design factors. This distinction supports the central concept of this study: the functional performance of a wearable microcurrent interface is governed not by intrinsic conductivity alone, but by the coupled design of conductive network continuity, spike architecture, compliant contact, volume-resistive behavior, and transient electrical response.

### 3.2. Geometry-Dependent Apparent Interfacial Resistive Response and Relative Current Redistribution

The developed conductive silicone interface consisted of three-spike and four-spike configurations, with each protrusion having a height of 7 mm and a representative diameter of 3.6 mm. Based on the geometrical model, the single-spike conductive area was 0.102 cm^2^, giving total effective conductive areas of 0.305 cm^2^ and 0.407 cm^2^ for the three-spike and four-spike structures, respectively. Relative to a planar contact area of 3 cm^2^, these values corresponded to analytical current-density amplification factors of 9.82 and 7.37, respectively. These calculated relationships indicate that the spike-structured conductive silicone should not be regarded simply as a passive soft conductor. Instead, the protrusion architecture changes the effective conductive area and therefore modifies the expected interfacial current-transfer behavior under the same total-current assumption.

**Figure 2:**
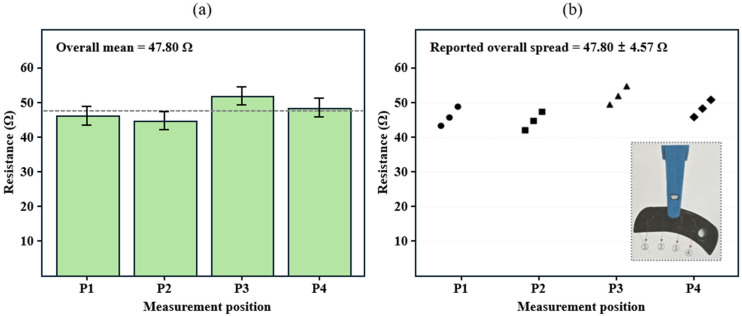
Resistance mapping of the conductive silicone interface. (**a**) Mean resistance values measured at four positions. (**b**) Raw resistance values showing the experimental spread of the conductive silicone interface. Error bars represent the standard deviation of triplicate measurements. The dashed line indicates the overall mean resistance, and different symbols represent repeated measurements at each measurement position.

The resistance mapping data support the practical reproducibility of this interpretation. The position-averaged resistance values were 46.03, 44.76, 51.97, and 48.45 Ω, and the 12 raw measurements yielded an overall mean of approximately 47.80 Ω with a standard deviation of approximately 4.57 Ω and a coefficient of variation of approximately 9.6%. This indicates measurable but acceptable positional variation for a soft elastomeric interface and confirms that the conductive silicone occupies an intermediate functional regime between metallic conduction and insulating polymer behavior at the electrode level. The positional resistance distribution and raw experimental spread are shown in Figure 2.

**Figure 3:**
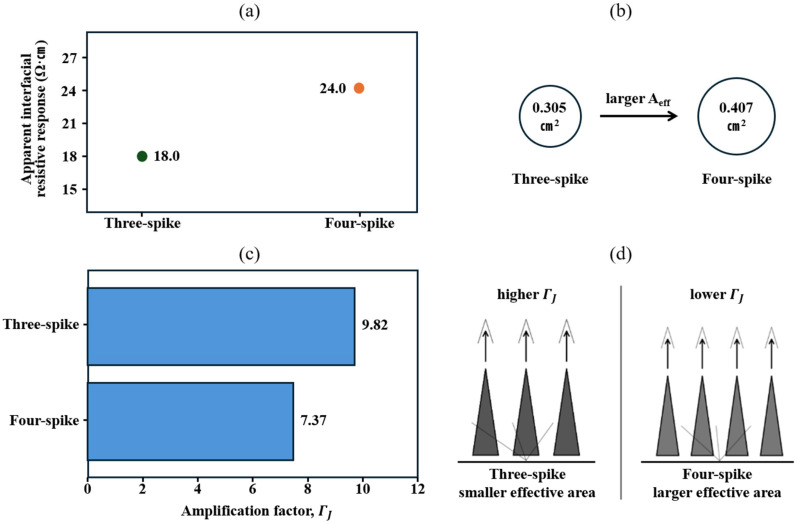
Geometry-dependent electrical interpretation of the spike-structured conductive silicone interface. (**a**) Apparent interfacial resistive response. (**b**) Total effective conductive area. (**c**) Estimated current-density amplification relative to a planar reference interface. (**d**) Conceptual effective-area-based comparison of the three-spike and four-spike configurations.

This geometry-derived interpretation is summarized in Table 2 and visualized in Figure 3, which compares the apparent interfacial resistive response, effective conductive area, and estimated current-density amplification for the two spike configurations. Using a fixed representative electrode-level resistance of 47.08 Ω, the geometry-derived apparent interfacial resistive response was estimated to be 18.0 Ω·cm for the three-spike structure and 24.0 Ω·cm for the four-spike structure. These values should be interpreted as analytical descriptors of the structured electrode assembly derived from the same measured representative resistance and different effective conductive areas, rather than as independently measured bulk resistivities, directly measured assembly resistivities, or experimentally mapped current distributions.

Accordingly, the distinction between the three-spike and four-spike configurations should be understood as a relative difference in effective area and expected current-transfer distribution under the same total-current assumption. It should not be interpreted as a direct field-mapping result, a PCB-resolved current simulation, or an experimentally measured local current-density distribution. In this context, Figure 3d serves only as a conceptual schematic of relative current redistribution within the continuous conductive silicone body. This point is important because the present study was designed to establish an effective-area-based analytical interpretation of two practical spike configurations, rather than a full parametric geometry optimization or field-distribution study.

Although increasing the number of protrusions might be expected to reduce resistance in an idealized simple parallel-resistor model, the present structure should not be interpreted solely on that basis. The spike array should instead be regarded as a continuous volume-resistive conductive elastomeric body in which the apparent electrode-level response may be influenced by pathway spacing, common conductive-body geometry, distributed current transfer through the soft conductive region, and interfacial spreading within the continuous silicone matrix. Accordingly, the change from three spikes to four spikes should be interpreted as a geometry-driven difference in effective conductive area and relative current-transfer behavior within the same continuous conductive silicone platform, rather than as discrete electrode-to-electrode switching behavior.

The present analysis therefore provides a structure–electrical interpretation of the molded conductive silicone interface under fixed material and dimensional conditions. A broader parametric optimization of spike height, spike diameter, pitch, base thickness, contact pressure, and frequency-dependent interfacial response remains necessary for future development of design rules for spike-structured conductive silicone interfaces.

### 3.3. Role of the Conductive Silicone Interface Under Low-Voltage Pulse Operation

The role of the conductive silicone interface should be interpreted in relation to the low-voltage pulse environment in which the material is used. The wearable microcurrent platform employs a 3.7 V-class Li-ion polymer battery and a compact control board connected to the conductive silicone contact region. Under this configuration, the conductive silicone does not function as the current-generating element itself. Instead, it serves as the terminal polymeric interface that transfers circuit-defined pulse signals toward the contact-facing spike region while maintaining elastomeric compliance and molded structural reproducibility.

**Figure 4:**
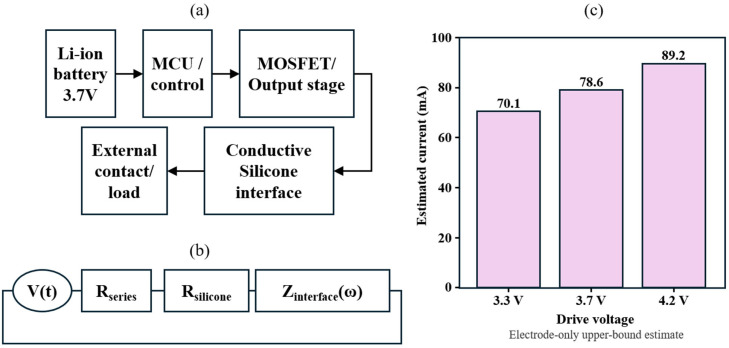
Low-voltage device-level coupling of the conductive silicone interface. (**a**) Simplified block diagram of the wearable microcurrent platform. (**b**) Equivalent circuit concept showing the conductive silicone interface as the terminal polymeric interfacial layer and an unspecified interfacial contribution. (**c**) Electrode-only Ohmic upper-bound estimate based on the representative electrode-level resistance. Note: The electrode-only upper-bound estimate is used only to illustrate that the conductive silicone body is not the dominant resistive element under the simplified electrode-only assumption and does not represent actual delivered stimulation current, human skin-contact impedance, or physiological current delivery.

From a materials perspective, the measured electrode-level resistance indicates that the conductive silicone body provides an electrically continuous pathway within the structured electrode. However, the conductive silicone should not be interpreted as a simple metallic conductor or wire-like component. Because it is a volume-resistive elastomer containing a distributed conductive network, its functional role is better understood as a soft interfacial medium that combines electrical continuity, compliant contact, geometry-defined current transfer, and transient electrical response. In this context, the low-voltage device-level coupling model in Figure 4 is used only to describe the position of the conductive silicone interface within the device current path. It is not intended to determine physiological current delivery, skin–electrode impedance, or stimulation efficacy.

Accordingly, the conductive silicone interface performs three coupled functions in the present system: (i) maintaining electrical continuity within the molded elastomeric body, (ii) preserving mechanical compliance during repeated contact, and (iii) enabling geometry-defined pulse transfer through the spike-structured contact region. These functions are particularly relevant for molded conductive silicone electrodes because their performance cannot be evaluated solely from intrinsic conductivity. Instead, the electrode-level response depends on the combined effects of conductive network continuity, volume-resistive behavior, spike geometry, contact configuration, and transient signal stability.

To further examine the effect of the conductive silicone body on pulse transfer, transient electrical response was evaluated under a controlled square-wave input. A 5 V, 1 kHz square-wave signal was applied, and the output waveform was recorded using a RIGOL DHO924S oscilloscope (RIGOL Technologies Co., Ltd., Suzhou, China) at a time scale of 20 μs/div. The waveform was compared under two conditions: without the conductive silicone interface and with the conductive silicone interface. This comparison was designed to evaluate whether the conductive silicone body distorted, attenuated, or damped rapid transient components during square-wave transmission.

In the condition without the conductive silicone interface, the square-wave signal was transmitted through a relatively direct electrical pathway. The waveform showed a sharp edge transition and a measurable overshoot, indicating that the fast switching component of the square-wave input was directly reflected in the output response. In contrast, after integration of the conductive silicone interface, the overall square-wave waveform was maintained, confirming that the conductive silicone did not severely suppress or distort the applied pulse signal under the tested condition. However, the edge transition and overshoot behavior were slightly moderated, indicating that the volume-resistive conductive silicone body affected the transient response of the pulse waveform.

In repeated waveform acquisitions, the overshoot values were measured as 3.5%, 3.4%, and 3.3% without the conductive silicone interface and 2.8%, 2.7%, and 2.6% with the conductive silicone interface. These values correspond to average overshoot values of 3.4 ± 0.1% and 2.7 ± 0.1%, respectively. The conductive silicone interface maintained the overall square-wave waveform while showing a modest reduction in overshoot under the tested condition. This reduction suggests that the conductive silicone interface can partially moderate rapid voltage transitions while preserving the overall square-wave waveform. This behavior is consistent with possible distributed resistive–capacitive contributions within the conductive elastomeric network. Therefore, the conductive silicone interface should be interpreted not as a simple low-resistance metallic pathway, but as a volume-resistive polymeric network that can influence transient pulse transfer.

The FFT displays obtained from the oscilloscope provided qualitative frequency-domain information for the measured square-wave responses. Both conditions showed frequency components associated with square-wave transmission, including the fundamental frequency and harmonic components arising from rapid rise and fall transitions. Because the absolute FFT amplitude can be affected by the output voltage level and oscilloscope display settings, the FFT response was used only as a qualitative waveform-monitoring display rather than as a quantitative impedance or harmonic attenuation analysis.

Importantly, this transient response analysis should not be interpreted as full electrochemical impedance spectroscopy, human skin–electrode impedance characterization, current-distribution simulation, or physiological validation. Instead, it provides conductive-silicone-centered experimental evidence showing how the molded conductive silicone polymer interface affects pulse transmission, overshoot behavior, edge transition, and high-frequency waveform components under controlled measurement conditions. From a polymers-focused materials perspective, this result is relevant because it links the volume-resistive nature of the conductive silicone matrix to the transient electrical response of the molded spike-structured interface.

**Figure 5:**
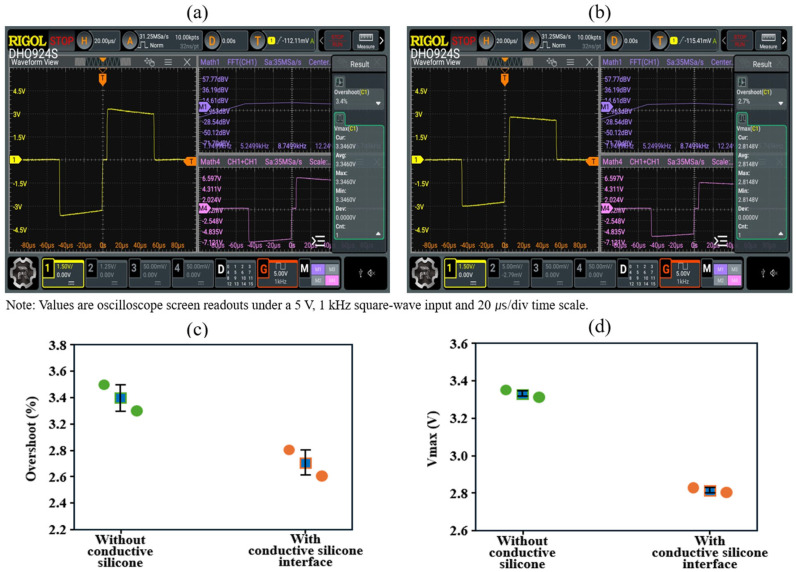
Transient electrical response under a 5 V, 1 kHz square-wave input. (**a**,**b**) Representative instrument-generated oscilloscope screenshots without and with the conductive silicone interface, respectively. (**c**,**d**) Repeated readouts of overshoot and $V_{\max}$, respectively. Green and orange symbols indicate measurements without and with the conductive silicone interface, respectively. Squares and error bars indicate mean ± SD (n = 3). Note: The FFT traces and oscilloscope screen readouts in (**a**,**b**) are instrument-generated displays and are shown as qualitative waveform-monitoring information obtained under a 5 V, 1 kHz square-wave input at a 20 μs/div time scale.

Therefore, the low-voltage pulse operation of the present system should be understood as a conductive silicone-centered interfacial phenomenon. The conductive silicone interface maintains pulse transfer while providing a soft, molded, and volume-resistive pathway whose response is governed by the coupled effects of conductive network formation, spike architecture, electrode-level resistance, and transient electrical behavior. The representative oscilloscope screenshots and repeated readouts of overshoot and Vmax are summarized in Figure 5. Further studies involving frequency-dependent impedance spectra, controlled contact media, skin-contact impedance, and physiological validation will be necessary to fully evaluate the electrode as a wearable stimulation interface.

### 3.4. Conductive Network Formation and Usability Trade-Off

The geometry-dependent and transient-response behaviors described above should be interpreted together with the conductive network behavior of the silicone elastomer itself. In conductive silicone systems, electrical continuity is generally achieved through the formation of a distributed conductive filler network within the elastomeric matrix. Therefore, the electrode-level response is not only governed by the nominal conductivity of the material, but also by the continuity of the conductive network, filler dispersion, matrix composition, surface condition, and molded structural integrity.

The supplementary formulation-dependent comparison showed that the measured resistance increased from 105 Ω to 145 Ω when the formulation condition shifted from a lower to a higher silicone-rich condition. This result indicates that increasing the silicone-rich fraction can dilute or disrupt the conductive network within the elastomeric body, thereby increasing the electrode-level resistance. Conversely, increasing the conductive carbon fraction lowered the measured resistance, but aggravated surface transfer or staining. This behavior reflects a typical trade-off in conductive elastomer systems: improving electrical transport by increasing conductive filler loading can be effective, but excessive filler exposure or weakly bound conductive domains may compromise surface cleanliness, practical usability, and repeated-contact stability.

This trade-off is particularly important for spike-structured conductive silicone interfaces because the material must simultaneously satisfy electrical, mechanical, and interfacial requirements. A lower resistance is beneficial for maintaining conductive continuity and pulse transfer, but maximum conductivity alone is not an appropriate design target. If the conductive filler content is increased excessively, the surface may become more prone to transfer, contamination, or unstable contact behavior. In contrast, increasing the silicone-rich fraction can improve elastomeric characteristics and surface cleanliness, but may reduce conductive network continuity and increase electrode-level resistance. Thus, the practical formulation window should be determined by balancing conductive network formation with elastomeric compliance, molded shape fidelity, surface stability, and device-level pulse-transfer behavior.

This interpretation also helps clarify the role of the commercial conductive LSR comparator. The primary conductive silicone rubber used in this study represents a relatively higher-conductivity conductive silicone platform suitable for molded spike-structured current transfer, whereas SILASTIC™ HV 1523-30 represents a softer conductive LSR class with higher reported volume resistivity. Because the conductive LSR comparator was not fabricated into the same spike-structured electrode and was not tested under identical experimental conditions, this comparison should be understood as a material-positioning and design-window discussion rather than as a direct paired performance comparison. Within this framework, the key design issue is not whether one commercial material is universally superior to another, but how the conductive silicone material class should be selected and managed according to the required balance between conductivity, softness, moldability, surface cleanliness, and interfacial stability.

When the formulation-level, structure-level, and transient-response observations are integrated, a clear design principle emerges. The functional performance of the spike-structured conductive silicone interface is determined by the coupled effects of conductive network continuity in the silicone matrix, volume-resistive behavior, molded spike geometry, electrode-level resistance, transient pulse response, and practical surface usability. The spike geometry controls the effective-area-based current-transfer behavior, whereas the conductive silicone formulation controls the conductive pathway continuity and usability of the molded body. Therefore, the present conductive silicone interface should be understood not simply as a soft conductive electrode material, but as a volume-resistive polymeric interface platform whose performance must be managed through both structural design and conductive network control.

**Figure 6:**
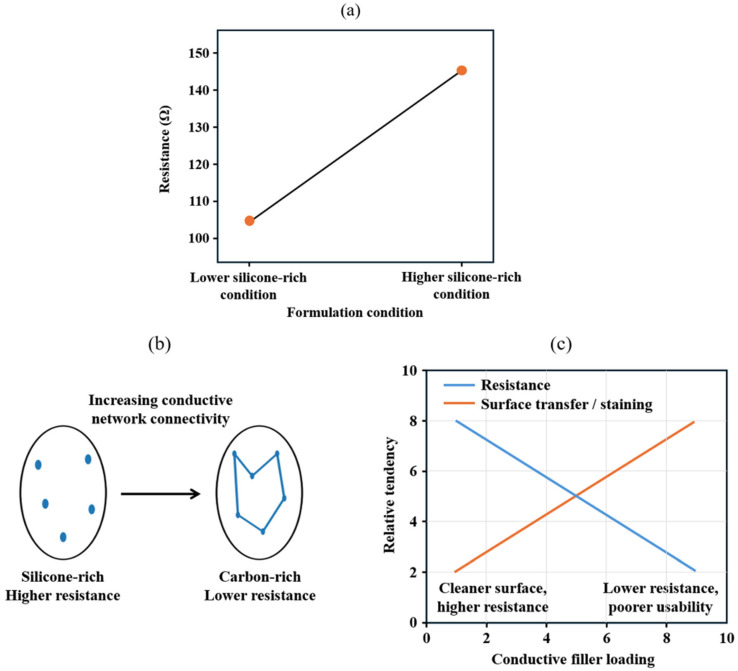
Formulation-dependent electrical behavior and usability trade-off of the conductive sili-cone system. (**a**) Resistance variation with formulation condition. (**b**) Conceptual conductive net-work evolution in the silicone matrix. (**c**) Schematic illustration of the trade-off between reduced resistance and increased surface transfer or staining with increasing conductive filler loading.

The formulation-dependent resistance behavior and the corresponding usability trade-off are summarized in Figure 6. Overall, these results suggest that future development of conductive silicone interfaces should not focus only on reducing resistance. Instead, the material should be optimized by considering resistance reproducibility, conductive filler dispersion, surface transfer, molding stability, contact durability, and transient electrical response together. Such a balanced design approach is essential for translating conductive silicone materials into reproducible wearable bioelectronic interfaces requiring compliant contact, localized pulse transfer, and stable repeated use.

## 4. Conclusions

In summary, this study demonstrates that the functional behavior of spike-structured conductive silicone interfaces is governed by the coupled effects of conductive network formation, volume-resistive behavior, molded spike geometry, electrode-level resistance, transient pulse response, and low-voltage device integration. The structured conductive silicone interface was fabricated using a commercially available electrically conductive two-component silicone rubber and implemented as the terminal polymeric interface of a wearable microcurrent platform. Within this framework, the conductive silicone should be interpreted not simply as a soft bulk conductor, but as a volume-resistive, geometry-defined current-transfer medium.

Based on the geometry-derived analytical model, the apparent interfacial resistive response was estimated to be 18.0 Ω·cm for the three-spike structure and 24.0 Ω·cm for the four-spike structure. The corresponding effective conductive areas of 0.305 cm^2^ and 0.407 cm^2^ gave analytical current-density amplification factors of 9.82 and 7.37, respectively, relative to a planar 3 cm^2^ reference interface. These values should be understood as effective-area-based analytical descriptors of the structured electrode assembly rather than as intrinsic bulk properties, directly measured current-density values, or field-mapping results. Positional resistance mapping further showed an overall mean resistance of 47.80 ± 4.57 Ω, indicating acceptable electrode-level reproducibility across the fabricated conductive silicone interface.

Transient response analysis under a 5 V, 1 kHz square-wave input further supported the conductive-silicone-centered interpretation. The conductive silicone interface maintained the overall pulse waveform while showing a modest reduction in overshoot from 3.4 ± 0.1% to 2.7 ± 0.1%. The FFT traces were used as qualitative waveform-monitoring displays rather than as quantitative impedance or harmonic attenuation data. These results suggest that the conductive silicone body behaves not as a simple metallic conductor, but as a distributed volume-resistive elastomeric network capable of influencing rapid transient components while preserving pulse transmission. The formulation-dependent comparison also showed that increasing the silicone-rich fraction increased the measured resistance from 105 Ω to 145 Ω, whereas increasing conductive carbon loading reduced resistance but aggravated surface transfer or staining, highlighting the need to balance conductive network continuity, surface cleanliness, elastomeric compliance, transient stability, and manufacturability.

Overall, this work establishes a practical structure–electrical property framework for spike-structured conductive silicone interfaces in wearable bioelectronic and electroceutical devices. The present study does not claim full electrochemical impedance characterization, skin–electrode impedance validation, current-distribution mapping, or physiological stimulation efficacy. Future studies should therefore investigate frequency-dependent impedance spectra, controlled contact media, skin-contact impedance, long-term repeated-use stability, and broader structural variables such as spike height, diameter, pitch, and contact pressure. Nevertheless, the present results provide useful guidance for designing and managing conductive silicone polymer interfaces that require compliant contact, reproducible geometry, and controlled pulse-transfer behavior.

## Figures and Tables

**Table 1:** Design window of the conductive silicone interface and comparative properties of the primary conductive silicone rubber and the conductive LSR comparator.

Category	Parameter	Primary Conductive Silicone Rubber	Conductive LSR Comparator
Material class	Type	Conductive silicone rubber	Conductive liquid silicone rubber
Commercial example	Product	ELASTOSIL^®^ R 573/70 A/B	SILASTIC™ HV 1523-30
Device role	Functional positioning	Structured conductive interface for localized current transfer	Softer commercial benchmark for material-class comparison
Electrical property	Volume resistivity (Ω·cm)	1.6	80
Mechanical property	Hardness (Shore A)	71	35
	Tensile strength (MPa)	6.2	5.6
	Elongation at break (%)	246	650
Interface design	Spike configuration	3-spike/4-spike	—
	Spike height (mm)	7	—
	Spike diameter (mm)	3.6	—
	Base thickness (mm)	1	—
	Effective current-path length (mm)	8	—

Note: Values for the commercial materials are based on manufacturer-reported catalog information and are used for material-class positioning, not for direct experimental comparison.

**Table 2:** Geometry-derived electrical parameters of the three-spike and four-spike conductive silicone interfaces.

Parameter	Unit	Three-Spike Interface	Four-Spike Interface
Number of spikes, *n*	—	3	4
Single-spike conductive area, $A_{\mathrm{single}}$	cm^2^	0.102	0.102
Effective conductive area, $A_{\mathrm{eff}}$	cm^2^	0.305	0.407
Effective current-path length, L	cm	0.8	0.8
Representative electrode-level resistance, $R_{\mathrm{rep}}$	Ω	47.08	47.08
Apparent interfacial resistive response, $\rho_{\mathrm{app}}$	Ω·cm	18.0	24.0
Planar reference area, $A_{\mathrm{planar}}$	cm^2^	3.0	3.0
Current-density amplification factor, $\Gamma_{J}$	—	9.82	7.37

## Data Availability

The original contributions presented in this study are included in the article. Further inquiries can be directed to the corresponding authors.
